# Insights into species-specific regulation of ANP32A on the mammalian-restricted influenza virus polymerase activity

**DOI:** 10.1080/22221751.2019.1676625

**Published:** 2019-10-14

**Authors:** Zhenwei Bi, Hongliu Ye, Xingbo Wang, An Fang, Tianqi Yu, Liping Yan, Jiyong Zhou

**Affiliations:** a MOE Joint International Research Laboratory of Animal Health and Food Safety, Institute of Immunology and College of Veterinary Medicine, Nanjing Agricultural University, Nanjing, People’s Republic of China; b MOA Key Laboratory of Animal Virology, Institute of Preventive Veterinary Sciences and Department of Veterinary Medicine, Zhejiang University, Hangzhou, People’s Republic of China; c Collaborative innovation center and State Key laboratory for Diagnosis and Treatment of Infectious Diseases, First Affiliated Hospital, Zhejiang University, Hangzhou, People’s Republic of China

**Keywords:** ANP32A, influenza A virus, polymerase activity, mammalian adaption, viral RNA

## Abstract

The ANP32A is responsible for mammalian-restricted influenza virus polymerase activity. However, the mechanism of ANP32A modulation of polymerase activity remains poorly understood. Here, we report that chicken ANP32A (chANP32A) -X1 and -X2 stimulated mammalian-restricted PB2 627E polymerase activity in a dose-dependent manner. Distinct effects of ANP32A constructs suggested that the ^180^VK^181^ residues within chANP32A-X1 are necessary but not sufficient to stimulate PB2 627E polymerase activity. The PB2 N567D, T598V, A613V or F636L mutations promoted PB2 627E polymerase activity and chANP32A-X1 showed additive effects, providing further support that species-specific regulation of ANP32A might be only relevant with the PB2 E627K mutation. Rescue of cycloheximide-mediated inhibition showed that ANP32A is species-specific for modulation of vRNA but not mRNA and cRNA, demonstrating chANP32A-X1 compensated for defective cRNPs produced by PB2 627E virus in mammalian cells. The promoter mutations of cRNA enhanced the restriction of PB2 627E polymerase in mammalian cells, which could be restored by chANP32A-X1, indicating that ANP32A is likely to regulate the interaction of viral polymerase with RNA promoter. Coimmunoprecipitation showed that ANP32A did not affect the primary cRNPs assembly. We propose a model that chANP32A-X1 regulates PB2 627E polymerase for suitable interaction with cRNA promoter for vRNA replication.

## Introduction

Facing multiple barriers to cross-species transmission, avian influenza A viruses (AIVs) increase their host range to infect new hosts via a high mutation rate, reassortment of genome segments and antigenic shifts [1]. Since 1997, highly pathogenic H5N1 virus infections have occurred in humans [2]. The pandemic 2009 H1N1 viruses were diversified by intermediate host pigs and caused new pandemics in humans [3]. In 2013, H7N9 AIV led to severe human disease and death in China. Furthermore, five epidemic waves of H7N9 infection in humans have occurred since then [4,5]. Additionally, increasing numbers of AIV subtypes, such as H9N2, H6N1, H10N8, H7N3 and H7N7, have sporadically occurred in humans [6–12]. This fact has raised serious concerns about the potential of AIV to cause a deadly pandemic similar to the 1918 Spanish influenza [13]. These outbreaks underscore the need to understand how AIV cross species barriers and develop infectivity in humans.

The influenza A virus genome is composed of eight single-stranded negative-sense RNA segments, which are coated by nucleoproteins (NPs) to form viral ribonucleoprotein complexes (vRNPs) with viral polymerase [14]. The viral polymerase consists of three proteins: PB1, PB2 and PA. PB1, as the core of the complex, constitutes a central RNA polymerase domain with the C-terminal domain of PA and the N-terminal domain comprising one-third of PB2. The N-terminal PA endonuclease domain and the C-terminal domain comprising two-thirds of PB2 formed several flexible peripheral appendices [14], and they undergo different conformational distributions for binding the conserved terminal ends of vRNA or cRNA [15]. Both transcription and replication of the influenza virus genome are catalyzed by the viral polymerase. The cap structure of cellular mRNA is recognized and bound by PB2, and the capped RNA is cleaved 10–15 bases downstream of the 5′-terminus of the mRNA by the endonuclease activity of PA. This cleaved, short RNA with a 5′-cap structure serves as a primer for the initiation of transcription by the resident polymerase in the vRNPs [16]. After elongation of the nascent RNA chains, the polymerase reaches the poly (U) stretch on the 5′ terminal ends of the vRNA template and slips repeatedly, leading to the addition of a poly (A) tail at the 3′ end of viral mRNA [17]. Replication of the viral genome takes place in a primer-independent manner and proceeds in two steps. In the first step, the resident polymerase in vRNPs synthesizes full-length complementary RNA (cRNA), which is incorporated into a replicative intermediate cRNP with newly synthetized PB1, PB2, PA and NP. This process starts from U(-1)C(-2) on the 3′ promoter of the vRNA template, which is called terminal initiation [14]. The second step of replication is internal initiation. It occurs at U(-4)C(-5) on the 3′ promoter of the cRNA and leads to the generation of a dinucleotide ApG primer that is used to realign at U(-1)C(-2) and primes full-length vRNA. vRNA replication from the cRNA depends on a second transactivating or transacting polymerase in addition to the resident polymerase in cRNPs [14,17].

Host restriction of AIV involves multiple factors, such as receptor preference, virion stability and polymerase activity. The viral polymerase plays an important role in virus replication, host specificity and pathogenicity. Multiple mutations have been identified in the PB2 subunit for enhancing polymerase activity in a mammalian-specific or nonspecific manner [18]. A well-characterized adaptive mutation is the substitution of the amino acid at position 627 of PB2 from the avian signature Glu (E) to the mammalian-adapted signature Lys (K). The PB2 E627K substitutions were rapidly selected upon infection of humans with H5N1 or H7N9 viruses as well as other subtypes of AIV [4,5,9], which have been associated with enhanced polymerase activity, high virus replication and pathogenicity in humans. The molecular mechanism of the PB2 E627K mutation in the upregulation of mammalian-restricted polymerase function has been explored for decades. The PB2 E627K mutation enhanced vRNP stability in mammalian cells, but the enhanced stability may be a result of the greater amount of vRNPs produced by the higher activity of the mammalian-adapted polymerase PB2 E627K than the avian-signature polymerase PB2 627E [19]. The restriction of the avian polymerase was overcome by a short viral RNA template or mutations specific to the 3′ promoter of the vRNA template, proposing that the PB2 627 residue is related to the viral promoter [20]. In addition, the PB2 627 residue has been linked with the interaction between the virus polymerase and host factors [21]. Human importin-α1 and -α7, which are required for the accumulation of vRNPs in the nucleus and efficient polymerase activity in human cells, bind more strongly to mammalian-adapted vRNPs (PB2 627K) than avian-signature vRNPs (PB2 627E) [21]. Human Tu elongation factor mitochondrial (TUFM) binds much more strongly to avian-signature PB2 627E than mammalian-adapted PB2 627K and impedes AIV replication in human cells in a manner that correlates with autophagy [22]. During virus infection, incoming avian vRNPs (PB2 627E) are directly impaired by RIG-I leading to virus inhibition, which is impeded by the PB2 E627K mutation. However, avian polymerase activity was not rescued in human cells lacking RIG-I [23]. The ANP32A is identified to be different among species and chicken ANP32A (chANP32A) with an additional 33 amino acids expressed in mammalian cells overcome AIV restriction compared with mammalian ANP32A [24,25]. Further, a SUMO-interacting-motif-like sequence of the additional 33 amino acids is crucial for avian ANP32A to promote PB2 627E polymerase activity [26]. The human ANP32A (huANP32A) directly plays an important role in the acquisition of the PB2 E627K substitution during adaptation of H7N9 AIVs to humans [27]. However, how ANP32A differentially regulates polymerase activity for host adaption is not well understood.

In this study, we identified the precise step of mammalian-restricted AIV polymerase, which was differentially regulated by chANP32A and huANP32A. Also, the effects of other mutations in PB2 and cRNA promoter mutation on regulation of ANP32A in polymerase activity were investigated.

## Materials and methods

### Cell culture, viruses, and antibodies

293T cells were maintained in Dulbecco’s modified Eagle’s medium (DMEM, Hyclone) containing 10% fetal calf serum (Sigma), and DF-1 cells were maintained in DMEM containing 10% fetal calf serum (MRC) at 37°C in 5% CO_2_ atmosphere. A/chicken/Zhejiang/A2013/2017 (H9N2) (H9N2 virus), A/Shanghai/02/2013 (H7N9) (H7N9 virus) and A/Puerto Rico/8/1934 (H1N1) (PR8 virus) were available in our laboratory. Anti-Flag mouse monoclonal antibody (F1804, Sigma), anti-Myc rabbit polyclonal antibody (R1208-1, Hangzhou HuaAn Biotechnology Co., Ltd), anti-GAPDH rabbit polyclonal antibody (ABPR001, Hangzhou Goodhere Biotechnology Co., Ltd), anti-NP mouse monoclonal antibody (IT-003-023M1, Cambridge biologics), anti-human ANP32A rabbit polyclonal antibody (D122870-0025, Sangon Biotech), anti-PB2 rabbit polyclonal antibody (GTX125926-S, GeneTex), HRP-anti-mouse goat polyclonal antibody (074-1806, KPL), HRP-anti-rabbit goat polyclonal antibody (074-1506, KPL) were purchased from commercial sources. Duck embryonic fibroblasts (DEFs) were prepared from duck embryos, which were purchased from Yangzhou Junhua Breeding Poultry Co., Ltd in China.

### Cloning and amplification of ANP32A transcripts

Specific primers for full-length ANP32A were used to amplify ANP32A by RT–PCR. The cDNAs of ANP32A were transcribed from RNA extracted from 293T cells and DF-1 cells. Using the specific primers: forward primer 5′-ATGGAGATGGGCAGACGG-3′ and reverse primer 5′-TTAGTCATCATCTTCTCCCTCA-3′ for human ANP32A (huANP32A) and primers 5′-ATGGACATGAAGAAAAGG-3′ and 5′-TTAGTCATCTTCATCTCC-3′ for chicken ANP32A (chANP32A), PCR products were cloned into pCAGGS expression vector.

In order to detect the content of ANP32A in avian cells, the following primers were designed. forward primer 5′-CCTCCCACAACTCACATACCTCG-3′ and reverse primer 5′-TTCATCTTCTACTACCTGAGCATCA-3′ for chANP32A; forward primer 5′-CCTCCCGCAACTCACATACCTC-3′and reverse primer 5′-TTCGTCTTCTACTACCTGAGCGTCA-3′ for duck ANP32A (duANP32A); forward primer 5′-TGTGCCGCTAGAGGTGAAATT-3′ and reverse primer 5′-TGGCAAATGCTTTCGCTTT-3′ for 18S rRNA as internal control. The amplified PCR products were separated by 8% PAGE gel at 75 V for 3 h.

### Plasmid construction

Eight segments of PR8 virus were cloned into pBD, which was used to produce vRNA and mRNA of each segment and generate IAV. Site-directed mutagenesis was created by the inverse PCR technique and confirmed by sequencing. The human polI-vNA-Firefly luciferase reporter plasmids and chicken polI-vNA-Firefly luciferase reporter plasmids were constructed as described previously [28]. The vRNA-luciferase reporter plasmid pPolI-vNA-luc (vNA-Luc) contains the luciferase-coding sequence in the antisense orientation flanked by the 5′ and 3′ untranslated regions of the NA gene segment from PR8, and the cRNA-luciferase reporter plasmid pPolI-cNA-luc (cNA-Luc) contains the luciferase-coding sequence in the sense orientation flanked by the 5′ and 3′ untranslated regions of the NA gene segment. The C3U and G8A mutation at 5′ promoter, A3G and U8C mutation at 3′ promoter of cRNA were created by the inverse PCR technique and confirmed by sequencing. The expression plasmids encoding polymerase PB1, PB2, PA and NP of H9N2, H7N9 and PR8 viruses were cloned into pCAGGS linearized with *Eco*R I and *Xho* I. Similarly, ANP32A was cloned into pCAGGS and *Renilla* luciferase expression plasmid pCAGGS-*Renilla* was constructed as an internal control. For detection of protein expression, the Flag or Myc were N-terminally added onto the pCAGGS expression plasmid.

### Polymerase assay

Polymerase activity analysis was performed by using a cell-based polymerase reconstitution with vNA-Luc or cNA-Luc as previously stated [24]. Briefly, 293T cells or DF-1 cells were seeded into a 24-well plate and transfected with plasmids PB1, PB2, PA, and NP (0.2 μg each/well) and vNA-Luc or cNA-Luc reporter (0.1 μg each/well) as well as *Renilla* expression control (0.1 μg each/well), using Exfect 2000 transfection reagent (Vazyme) according to manufacturer’s instruction. Cells were incubated at 37°C for 24 h, lysed with 100 μL of Passive Lysis Buffer (Beyotime), and Firefly and *Renilla* luciferase bioluminescence was detected with an Infinite 200 PRO (TECAN). The polymerase activity was calculated as the activity of the Firefly luciferase normalized to that of the *Renilla* luciferase. The effect of ANP32A on influenza polymerase activity was examined by a polymerase assay after expression of ANP32A (0.5 μg/well) and PB1, PB2, PA, and NP (0.1 μg each/well), vNA-Luc or cNA-Luc and *Renilla* expression control (0.05 μg each/well) for 24 h.

### Generation and growth curve analysis of recombinant viruses

The PB2 K627E substitution of pBD-PB2 was performed by site-directed mutagenesis by PCR. The recombinant PR8 viruses carrying PB2 627K or K627E were rescued in 293T cells in the 8-plasmid system by the reverse genetics technique [29]. The progeny viruses were harvested at 48 h posttransfection and were inoculated into 10-day-old embryonated chicken eggs. The recombinant virus was confirmed by sequencing and its growth curve analysis was performed by infecting 293T cells with PR8-PB2 K627E or PB2 627K virus (MOI = 0.01). 293T cells were transfected with chANP32A-X1 (0.5 μg/well) using Lipofectamine 2000 (Invitrogen) for 24 h, infected with PR8-PB2 K627E virus for 1 h at 37°C (MOI = 0.01) and cultured for indicated time point. The virus titre was detected by Reed-Muench method using MDCK cells.

### RNA isolation, reverse transcription, and quantification by RT–PCR

Total RNA from infected or transfected 293T cells was extracted using TRIzol (Vazyme, China) according to the manufacturer’s instructions. The RT primers for differentiating vRNA, cRNA and mRNA of influenza virus were designed according to the reference [30] as follows: primer 5′-GACGATGCAACGGCTGGTCTG-3′ for the vRNA of NP, 5′-AGTAGAAACAAGG-3′ for the cRNA of NP, oligo(dT)20 (5′-TTTTTTTTTTTTTTTTTTTT-3′) for the viral mRNA, and random hexamers for GAPDH. Equal concentrations of RNA (1 μg) were subjected to cDNA synthesis using a ReverAid First Strand cDNA Synthesis Kit (Thermo) with specific primers or random hexamers (Thermo) according to the instructions. The cDNAs were subjected to quantification by real-time PCR using the FastStart SYBR Green Master (Roche), and the NP-specific primer set and GAPDH-specific primer set as follows: 5′-GACGATGCAACGGCTGGTCTG-3′ and 5′-AGCATTGTTCCAACTCCTTT-3′ for PR8-NP; 5′-GTCAGCCGCATCTTCTTTTG-3′ and 5′-GCGCCCAATACGACCAAATC-3′ for GAPDH. Real-time PCR was performed using a LightCycler 96 (Roche). Fold change of RNA levels compared with the empty vector was calculated by the 2^-ΔΔCT^ method, including normalization to CT values of GAPDH.

### Western blotting

Cells were lysed with Tris-Glycine SDS sample buffer (Invitrogen), heated for 10 min at 95°C, and then separated by SDS-PAGE and transferred onto nitrocellulose (NC) membranes. The membranes were blocked with 5% nonfat milk powder in PBS and then incubated with primary antibody and HRP-conjugated antibody. Then, protein bands on membranes were detected with ECL (Thermo).

### Coimmunoprecipitation assay

293T cells were cotransfected with Myc-tagged PB2 627E or 627K, PB1 or PB1 (D446Y), PA and NP as well as ANP32A and cNA-Luc template for 24 h. The cells were resuspended in NP-40 buffer (Beyotime) and incubated for 1 h at 4°C. Immunoprecipitations were performed with protein A/G-agarose (Santa Cruz) and 5 μg of mouse anti-NP monoclonal antibody. Immunoprecipitated proteins were washed and dissolved in SDS sample buffer, and the interaction between NP and polymerase was analyzed by western blot using mouse anti-NP monoclonal antibody and anti-Myc and anti-Flag antibodies (Sigma) for detection of PB2 and ANP32A, respectively.

## Results

### Amplification and sequencing of ANP32A

**Figure 1.**
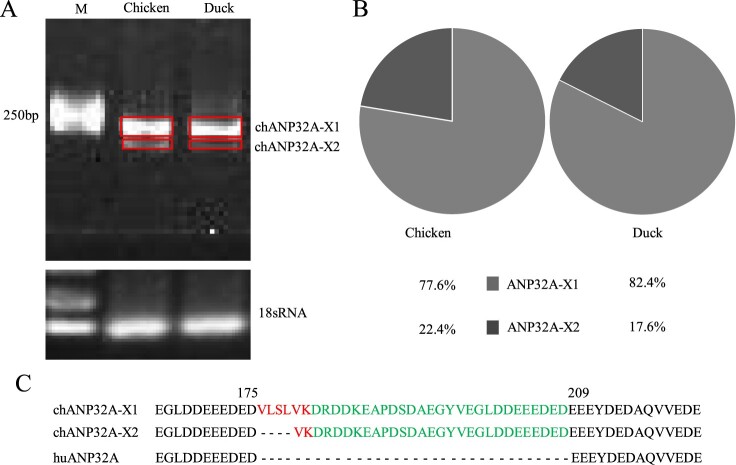
The relative abundance and sequence alignment of ANP32A. (A) The ANP32A in chicken and duck was amplified by RT-PCR. (B) The relative abundance of ANP32A is analyzed by IMAGE J and indicated by the pie charts. (C) Amino acid sequence alignment of ANP32A in chicken and duck. The SIM sequence (red) and 27 amino acid residues (green) are highlighted.

Species-specific differences of ANP32A affect the activity of the influenza polymerase during infection [24]. We cloned two different sizes of ANP32A from DF-1 cells with ANP32A-specific primers (Figure 1(A)). Sequencing indicated that the nucleotide length was 846 and 834 bp for the two ANP32As, which were the same sizes as the predicted *Gallus gallus* ANP32A transcript variants X1 (XM_413932) and X2 (XM_004943928) in GenBank, respectively, and named as chANP32A-X1 and chANP32A-X2. The relative abundance of two avian ANP32A-X1 and ANP32A-X2 were 77.6% and 22.4% in DF-1 cells, and 82.4% and 17.6% in DEFs by analysis of Image J software, respectively (Figure 1(B)). Chicken and duck ANP32A-X1 is demonstrated to have higher abundance in comparison with ANP32A-X2, which is similar to a recent result [25]. Additionally, human ANP32A (huANP32A) of 750 bp length was obtained from 293T cells. Alignment of the amino acid sequences revealed a 33 amino acid insertion of ^176^VLSLVKDRDDKEAPDSDAEGYVEGLDDEEEDED^208^ in chANP32A-X1 and a 29 amino acid insertion of ^180^VKDRDDKEAPDSDAEGYVEGLDDEEEDED^208^ in chANP32A-X2 in comparison with huANP32A (Figure 1(C)). The additional 33 amino acid insertion at position 175 in chANP32A-X1 comprises a repeat of 27 amino acids (^149^DRDDKEAPDSDAEGYVEGLDDEEEDED^175^) and 6 unique amino acids (^176^VLSLVK^181^), the ^176^VLSLV^180^ of which resembles a SUMO interaction motif-like sequence (SIM) [26]. The chANP32A-X2 lacks four hydrophobic amino acids (^176^VLSL^179^) from the N terminus of the SIM (Figure 1(C)).

### Different regulation of ANP32A in PB2 627E polymerase activity

**Figure 2.**
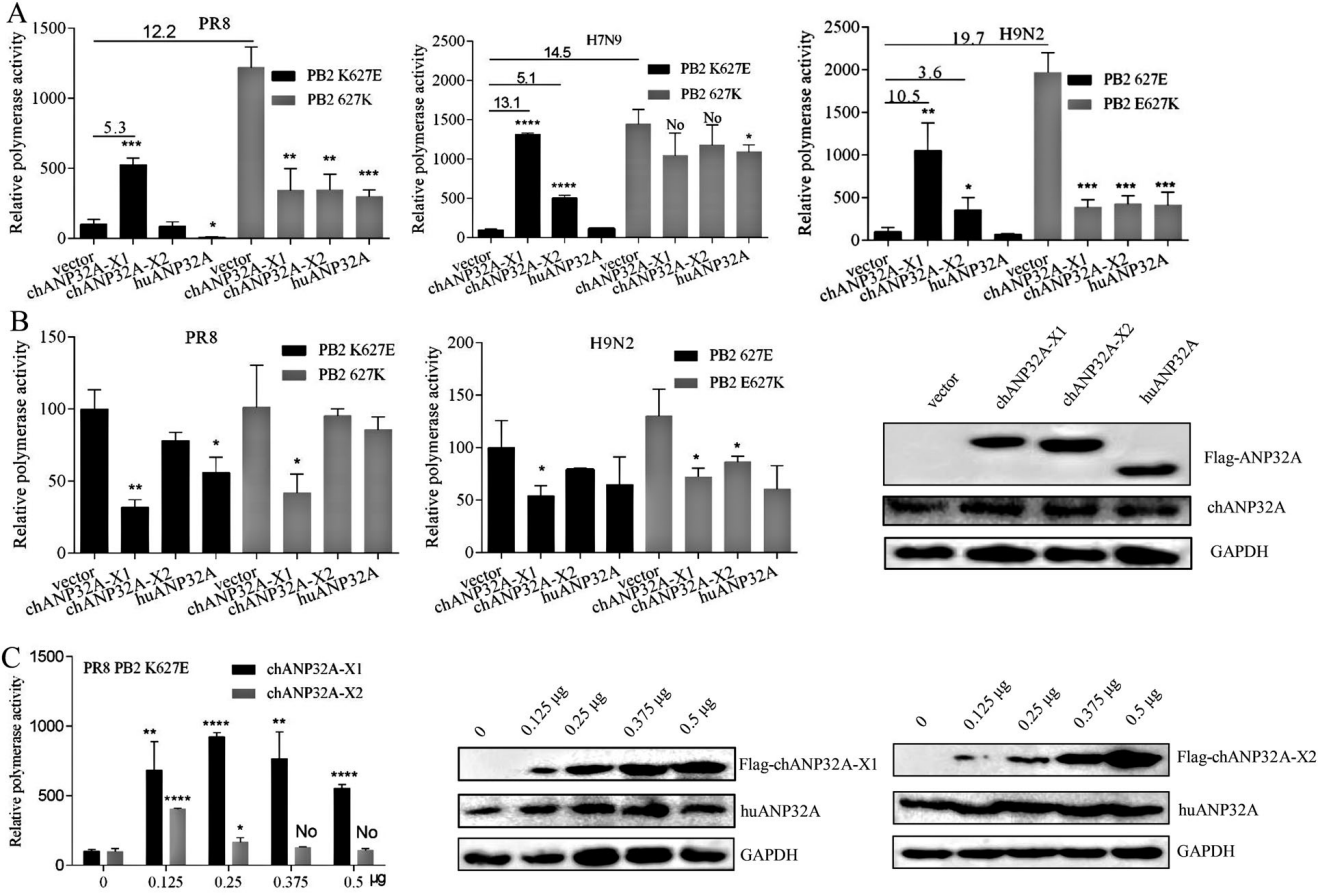
Species-specific regulation of PB2 627E polymerase activity by different ANP32A. (A-B) Effects of ANP32A on different influenza virus polymerase activities in 293T cells (A) and DF-1 cells (B). (C) The effects of different expression levels of chANP32A-X1 and chANP32A-X2 on the activity of PR8 PB2 K627E polymerase in 293T cells. Polymerase activity was detected using a Dual Luciferase Reporter Gene Assay Kit. Error bars represent one standard deviation (N = 3; ****p < 0.0001, ***p < 0.001, **p < 0.01, and *p < 0.05). (B-C) The expression of chANP32A-X1, chANP32A-X2 and huANP32A were detected by anti-Flag antibody; the endogenous chANP32A and huANP32A were detected by anti-huANP32A polyclonal antibody; the GAPDH were detected by anti-GAPDH polyclonal antibody.

The polymerase (PB1, PB2, PA) and NP from the avian-origin H9N2 and human-origin PR8 (H1N1) and H7N9 strains were cloned into pCAGGS vector, and the inverse PCR technique was used to introduce E or K mutations at the 627 position of PB2. Polymerase activity was assayed using cell-based reconstituted polymerase with the vRNA-like luciferase reporter gene drived by human PolI(vNA-Luc). Avian-signature PB2 627E polymerase activity derived from PR8, H9N2 and H7N9 strains was heavily restricted in 293T cells, but it was significantly activated in 293T cells expressing chANP32A-X1. Another chicken transcript, chANP32A-X2, increased the PB2 627E polymerase activity of H9N2 and H7N9 strains but could not stimulate PR8-derived PB2 K627E polymerase activity (Figure 2(A)). Further, we observed that the low level of chANP32A-X2 promoted PR8 PB2 K627E polymerase activity and overdose expression had a negative effect. The expression of chANP32A-X1 in 293T cells also supported PR8 PB2 K627E polymerase activity in a dose-dependent manner (Figure 2(C)). However, the support of chANP32A-X1 to the function of PB2 627E polymerase activity was stronger than chANP32A-X2 (Figure 2(A, C)), indicating that the N-terminal ^176^VLSL^179^ in the SIM of chANP32A-X1 is necessary for supporting its optimal modulation of PB2 627E polymerase activity. Consistently, when huANP32A was overexpressed, PB2 K627E polymerase activity of PR8 was significantly inhibited in 293T cells, while the H9N2 and H7N9 strains were not significantly affected, showing strain-specific. Interestingly, the PB2 627K polymerase of PR8 and H9N2 viruses exhibited inhibited activity in chANP32A-X1, chANP32A-X2 and huANP32A-overexpressing 293T cells. The chANP32A-X1 and chANP32A-X2 did not significantly affect the PB2 627K polymerase activity of H7N9, but the activity was inhibited by huANP32A in 293T cells (Figure 2(A)). These observations are consistent with earlier reports [24], demonstrating that ANP32A was species-specific for PB2 627E polymerase but not PB2 627K polymerase activity modulation of influenza virus in 293T cells. In contrast to results performed in 293T cells, PB2 627E or 627K polymerase displayed approximately equivalent levels of activity when assays were performed with the vRNA-like luciferase reporter gene drived by chicken PolI(vNA-Luc) in DF-1 cells, and none of the different ANP32A could stimulate activity for PB2 627E and 627K polymerase. Conversely, expression of chANP32A-X1 seemed to have a more significant inhibition of polymerase activity than the other ANP32A variants in DF-1 cells (Figure 2(B)). Collectively, these data suggested that PB2 627K polymerase was efficient at using both chANP32A and huANP32A, while the PB2 627E polymerase utilized chANP32A more effectively than huANP32A for its polymerase activity.

### The residues ^180^VK^181^ within the 33 aa insertion are necessary for species-specific modulation of ANP32A on PB2 627E polymerase activity

**Figure 3.**
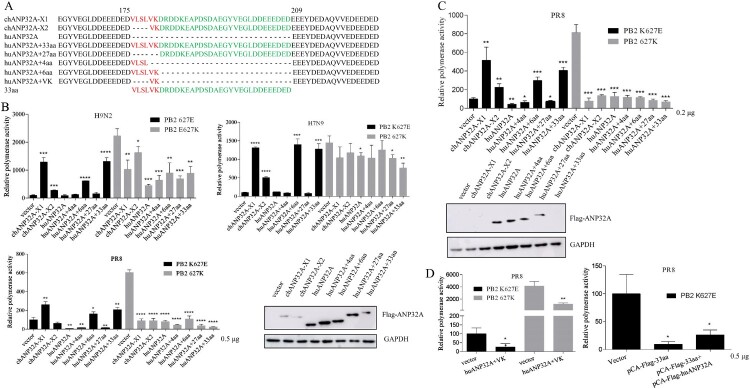
Effects of different chimeric ANP32A constructs on polymerase activity. (A) The amino acid sequence alignment of chANP32A-X1, chANP32A-X2, huANP32A and huANP32A constructs including 4, 6, 27 or 33 amino acid insertions. The SIM sequence (red) and 27 amino acid residues (green) are highlighted. (B-D) Polymerase activity assays were performed in the presence of different chimeric ANP32A constructs. Error bars represent one standard deviation (N = 3; ****p < 0.0001, ***p < 0.001, **p < 0.01, and *p < 0.05). (B-C) The expression of different chimeric ANP32A constructs in 293T cells was detected by anti-Flag antibody and the GAPDH were detected by anti-GAPDH polyclonal antibody.

To further identify the critical functional domains of the 33 amino acid insertion of chANP32A-X1 that are responsible for the enhanced PB2 627E polymerase activity, several chimeric huANP32A mutants were constructed (Figure 3(A)). As shown in Figure 3(B), huANP32A harbouring the N-terminal ^176^VLSLVK^181^ and the 33 aa insertion promoted PB2 627E polymerase activity of H9N2, PR8 and H7N9 strains, similar to chANP32A-X1. However, huANP32A containing the N-terminal ^176^VLSL^179^ and the 27 aa insertion without the SIM, similar to huANP32A, abrogated the ability to support PB2 627E polymerase activity of the three strains. The chANP32A-X2, with a 29 aa insertion, partially increased PB2 627E polymerase activity of H9N2 and H7N9 but not PR8 strain. Intriguingly, all of these ANP32A mutants were more harmful to PB2 627K polymerase activity of PR8 and H9N2 than that of H7N9. Considering that high levels of chANP32A had a negative effect on stimulating PB2 627E polymerase activity (Figure 2(C)), we tested the low expression levels of huANP32A, huANP32A containing ^176^VLSL^179^ and the 27 aa insertion did not still promote PB2 627E polymerase activity, except for chANP32A-X2 (Figure 3(C)). Under the condition of equal plasmid transfections, the expression levels of chANP32A-X1, chANP32A-X2 and huANP32A-33 are much lower than other human mutants in western blot assay (Figure 3(B,C)). However, huANP32A only containing ^180^VK^181^ or coexpression of huANP32A and the 33 aa could not promote PB2 627E polymerase activity (Figure 3(D)). These data demonstrated that the ^180^VK^181^ residues within the 33 aa insertion of chANP32A-X1 were the critical motif but not sufficient for maintaining the species-specific modulation of PB2 627E polymerase activity of influenza virus in 293T cells.

### Effect of PB2 mutation on the regulation of polymerase activity by ANP32A

**Figure 4.**
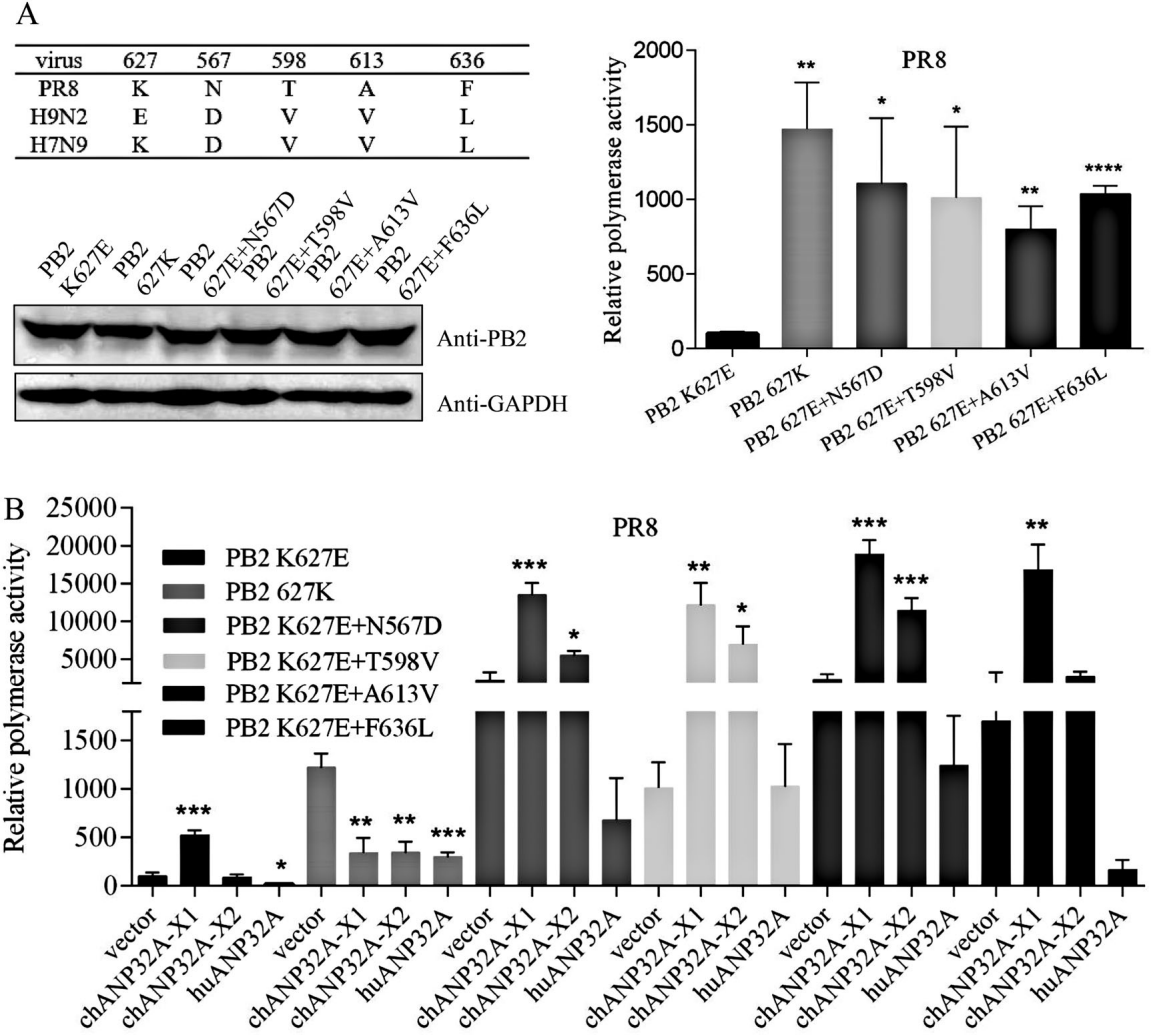
Effect of PB2 mutations on PR8 PB2 K627E polymerase and the regulation of ANP32A in PR8 PB2 K627E polymerase activity. (A) Comparison of mutant sites of PR8 PB2 627 domain with H9N2 and H7N9 and effect of PR8 PB2 mutations on polymerase activity. The expression of mutant PR8 PB2 was detected by anti-PB2 polyclonal antibodies in western blot. (B) Effect of PB2 mutations on the regulation of ANP32A in PR8 PB2 K627E polymerase activity. Error bars represent one standard deviation (N = 3; ****p < 0.0001, ***p < 0.001, **p < 0.01, and *p < 0.05).

The species-specific regulation of ANP32A in polymerase is relevant with the 627 site that located within the residues 535aa-667aa of PB2 627 domain. The 627 domain of PB2 is essential for the replication of RNPs in a cellular context [31]. We wanted to test whether other amino acid sites in the PB2 affect polymerase activity and ANP32A function. Compared with H9N2 and H7N9 strains, PR8 exhibited the D567N, V598T, V613A and L636F mutations (Figure 4(A)). The 598V and 636L are AIV signatures and the substitution of V598T and L636F increased polymerase activity in mammalian cells, respectively [18,32]. On the basis of PB2 K627E mutation, we performed the mutation of N567D, T598V, A613V and F636L for PR8 virus, respectively. These mutations significantly promoted the PB2 K627E polymerase activity although western blot showed the mutant PB2 had the similar expression levels (Figure 4(A)). Curiously, the mammalian adaptive amino acids T598 and F636 were mutated into avian virus signal amino acids, also increasing polymerase activity. Being different from other influenza strains, PR8 virus acquired multiple mutations highly adapted to mammalian cells, which may contribute to the increased polymerase together with T598V or F636L mutation. Overexpression of chANP32A-X1 further promoted the activity of polymerase with these mutants (Figure 4(B)). The additive facilitations reveals that chANP32A-X1 and the mutation of N567D, T598V, A613V and F636L promote PB2 627E polymerase in different manners. By comparison, PB2 E627K mutation promoted polymerase activity in 293T cells, but chANP32A-X1, chANP32A-X2 and huANP32A inhibited PB2 627K polymerase activity. Thus species-specific regulation of ANP32A in polymerase activity might be only related with PB2 E627K mutation. In addition, high level of chANP32A-X2 could not promote PR8 PB2 K627E polymerase activity (Figure 2(A,C)), but it could be promoted by chANP32A-X2 using the PB2 K627E protein with the three mutations of N567D, T598V or A613V, respectively (Figure 4(B)). One possibility is that the mutations of the three sites near to 627 site had an effect on PB2 K627E site, affecting the weak regulation of chANP32A-X2 in PB2 K627E polymerase.

### Species-specific regulation of ANP32A on vRNA but not mRNA and cRNA levels

**Figure 5.**
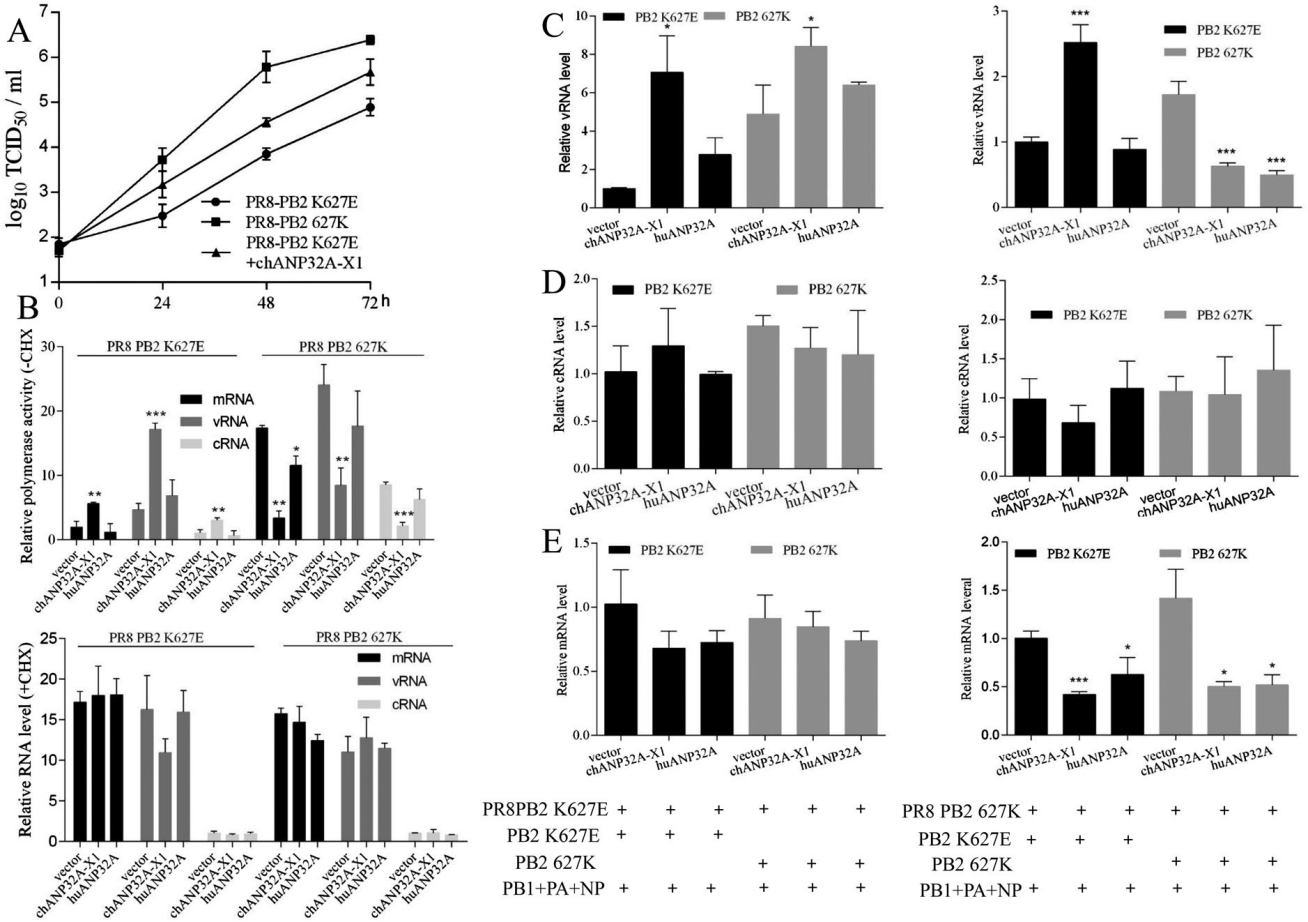
ANP32A differentially regulates vRNA levels. (A) The viral titres of PR8-PB2 K627E and PR8-PB2 627K in 293T cells. Wild-type or chANP32A-X1-expressed 293T cells were infected with PR8-PB2 K627E and PR8-PB2 627K (MOI = 0.01). After incubating for 1 h at 37°C, the cell supernatants were replaced with DMEM supplemented with 2% FCS and harvested every 24 h after infection for 72 h. The virus titres (TCID_50_) were determined at the indicated time points by Reed-Muench method using MDCK cells. (B) Effects of ANP32A overexpression on mRNA, vRNA and cRNA transcripts during influenza virus infection. 293T cells infected with either PR8-K627E or PR8-627K virus with or without 100 μg/mL cycloheximide (CHX) at an MOI of 5. Cells were collected at 4 h after infection, and mRNA, cRNA and vRNA levels were determined by qPCR. Cellular GAPDH served as an internal loading control. (C-E) Effects of ANP32A on vRNA (C), cRNA (D) and mRNA (E) levels during influenza virus infection with 100 μg/mL cycloheximide (CHX). 293T cells were transfected with PB1, PB2 627E or 627K, PA and NP (0.1 μg/well) as well as ANP32A (0.5 μg/well) at 37°C for 24 h, subsequently infected with the PR8-627K virus or PR8-K627E virus at an MOI of 5 and treated with CHX (100 μg/ml) for 4 h. The levels of vRNA, mRNA and cRNA were detected by qPCR at 4 h postinfection. Error bars represent one standard deviation (N = 3; ****p < 0.0001, ***p < 0.001, **p < 0.01, and *p < 0.05).

Compared with chANP32A-X2 and huANP32A, chANP32A-X1 strongly increased PB2 627E polymerase activity in 293T cells. However, it remains unclear whether this effect is a result of a promotion in transcription or replication or a combination of the two. To examine the effect of ANP32A on transcription and replication, the recombinant PR8 virus containing PB2 K627E (PR8-PB2 K627E) was generated in 293T cells by a plasmid-based reverse genetic system. In 293T cells, the viral titre of PR8-PB2 627K strain was higher than that of mutant PR8-PB2 K627E strain and chANP32A-X1 promoted the viral titre of mutant PR8-PB2 K627E strain (Figure 5(A)).

293T cells were transfected with chANP32A-X1 or huANP32A for 24 h, and then infected with PR8-PB2 K627E or PR8-PB2 627K virus at an MOI = 5 and cultured for 4 h in DMEM with or without 100 μg/mL cycloheximide (CHX). The qRT-PCR assay showed that in the absence of CHX chANP32A-X1 promoted level of mRNA, vRNA and cRNA for PR8-PB2 K627E virus but not PR8-PB2 627K virus. Comparatively, in the presence of CHX, PR8-PB2 K627E and PR8-PB2 627K viruses exhibited similar mRNA levels in 293T cells or 293T cells expressing chANP32A-X1 or huANP32A (Figure 5(B)), indicating that overexpression of chANP32A-X1 and huANP32A have no remarkable influence on primary mRNA transcription. Also, the newly synthesized cRNA could not be stabilized by viral proteins (PB1, PB2, PA and NP), and got rapid degradation [33], while the vRNA levels implied the initial incubated viruses.

To further determine whether the vRNA levels were regulated by ANP32A, we pre-expressed PB1, PB2 K627E or 627K, PA and NP as well as chANP32A-X1 or huANP32A in 293T cells for 24h before infection with PR8-PB2 K627E or PR8-PB2 627K virus (MOI = 5) and CHX-treatment for 4 h. In 293T cells infected with the PR8-PB2 K627E virus, chANP32A-X1, but not huANP32A, produced similar vRNA for both pre-expressed PB2 K627E and PB2 627K polymerase (Figure 5(C)). A previous study indicated that avian influenza virus (PB2 627E) in human cells synthesizes defective cRNPs, which are not bona fide templates for further vRNA synthesis by PB2 627E or PB2 627K polymerase [34]. Our data suggested that chANP32A-X1 compensated for the defective cRNPs for both pre-expressed PB2 K627E and 627K polymerase to produce vRNA. Comparatively, human influenza virus (PB2 627K) provides competent cRNPs for both pre-expressed PB2 K627E and PB2 627K polymerase to synthesize substantial amounts of vRNA in human cells [34]. We found that the pre-expressed PB2 627K polymerase seemed to produce more vRNA than PB2 K627E polymerase in 293T cells infected with the PR8-627K virus. Furthermore, chANP32A-X1, but not huANP32A, increased the amount of vRNA in cells pre-expressing PB2 K627E polymerase, while chANP32A-X1 and huANP32A inhibited the vRNA level in cells pre-expressing PB2 627K polymerase (Figure 5(C)). It is tempting to speculate that in the presence of pre-expressed PB2 627E polymerase in 293T cells, human influenza virus (PB2 627K) still produces defective cRNPs, which can still be further rescued by chANP32A-X1. When the E627K mutation occurs in pre-expressed PB2 polymerase, human influenza virus (PB2 627K) leads to optimal cRNPs, and the overexpression of chANP32A-X1 or huANP32A will be harmful. Simultaneously, the detection of cRNA levels showed that chANP32A-X1 and huANP32A did not significantly affect the cRNA levels (Figure 5(D)), suggesting that the species-specific regulation of ANP32A on vRNA was irrelevant to the amounts of cRNA. Although inhibition of the mRNA transcription of the PR8-627K virus was observed, chANP32A-X1 and huANP32A did not show species-specific effects on the mRNA level (Figure 5(E)). These data suggested that ANP32A differentially regulates vRNA levels for species-specific effects on PB2 627E polymerase activity.

### ANP32A differentially regulates polymerase activity from the cRNA promoter

**Figure 6.**
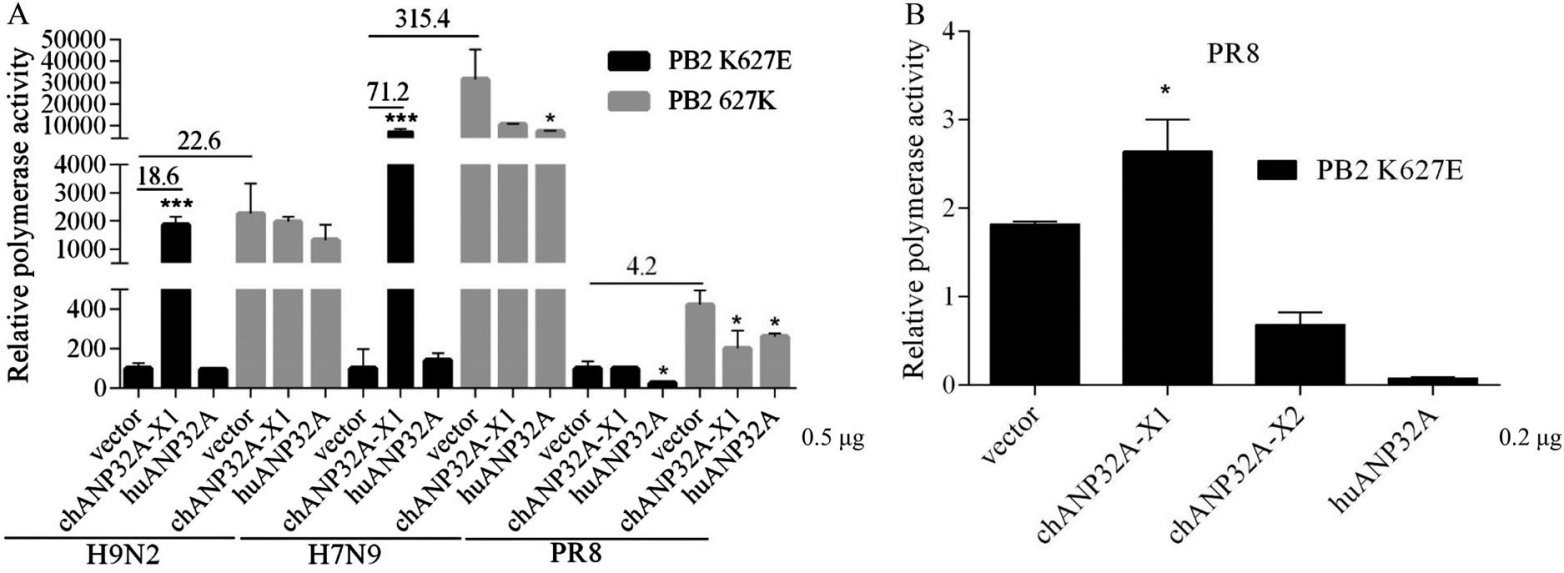
ANP32A differentially regulates polymerase activity from the cRNA promoter. (A) Effects of ANP32A on polymerase activity from the cRNA promoter for different influenza virus polymerases. (B) Effects of ANP32A on PR8-derived PB2 627E polymerase activity from the cRNA promoter. Luciferase activity was assayed. Error bars represent one standard deviation. (N = 3; ****p < 0.0001, ***p < 0.001, **p < 0.01, and *p < 0.05).

We analyzed the effects of ANP32A on polymerase activity from the cRNA-like luciferase reporter gene drived by human PolI(cNA-Luc) in cell-based polymerase reconstitution assays. The chANP32A-X1 and huANP32A were coexpressed with PB1, PB2 K627E or 627K, PA and NP as well as cNA-Luc and *Renilla* reporter control. As shown in Figure 6(A), the PB2 627E polymerase activity of H9N2 virus was 18.6-fold higher in 293T cells expressing chANP32A-X1 than in cells expressing the empty vector, whereas chANP32A-X1 had less stimulatory effect (approximately 10.5-fold higher) on the vNA-Luc promoter (Figure 2(A)). The PB2 K627E polymerase activity of H7N9 was also greatly increased by 71.2-fold in 293T cells expressing chANP32A-X1 using the cNA-Luc template, while only a 13.1-fold enhancement was observed in polymerase reconstitution assays with vNA-Luc template (Figure 2(A)). Interestingly, chANP32A-X1 did not show the ability to promote PB2 K627E polymerase activity for the PR8 virus, which was different from the results of the polymerase reconstitution assay with vNA-Luc as a template (Figure 2(A)). All of the ANP32A variants did not increase PB2 627K polymerase activity (Figure 6(A)). However, the reduced expression of chANP32A-X1 increased PR8 PB2 627E polymerase activity (Figure 6(B)). Collectively, ANP32A had a species-specific role in regulating PB2 627E polymerase activity from the cRNA promoter in human cells.

### Effects of cRNA promoter mutations on ANP32A function with respect to polymerase activity

**Figure 7.**
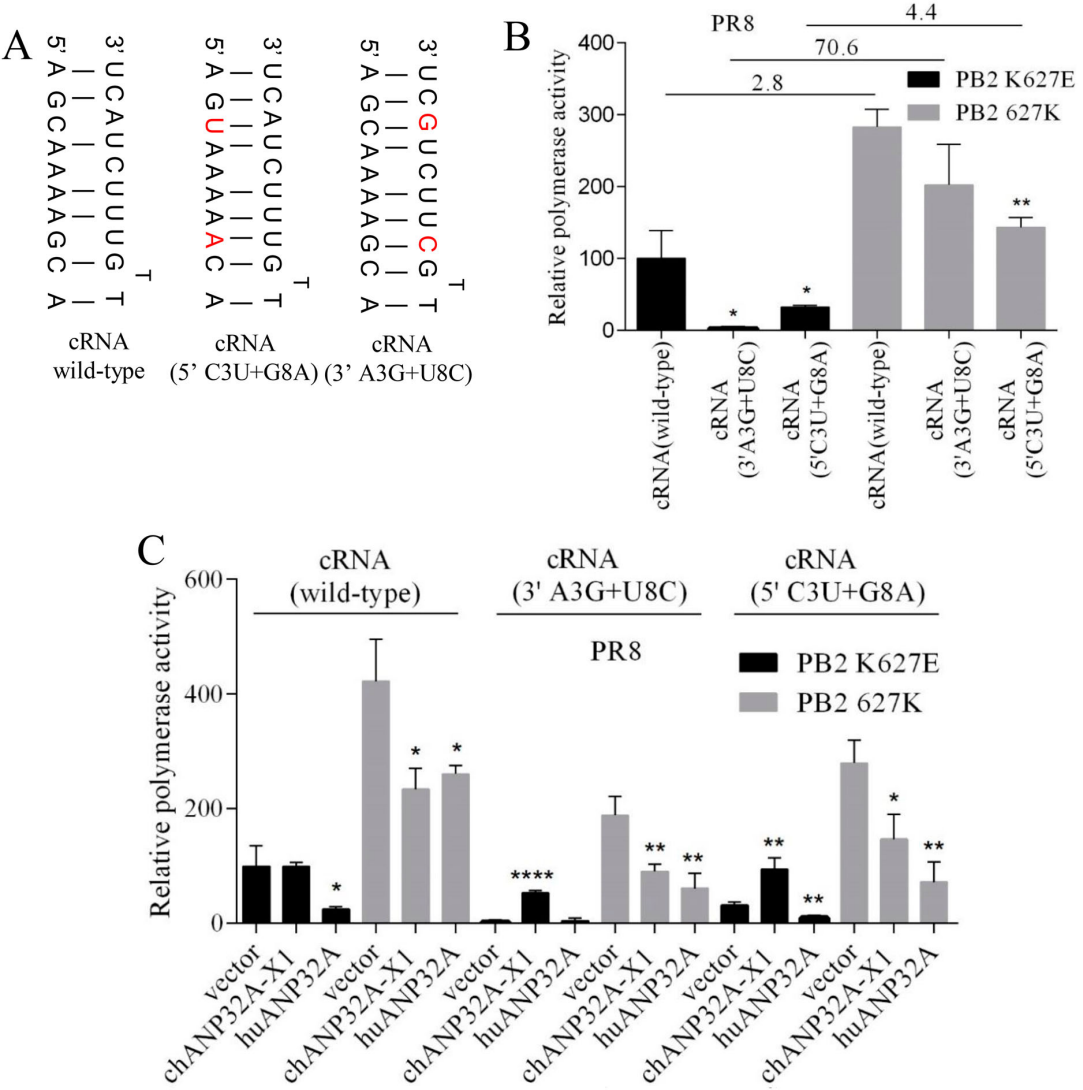
Effects of promoter mutation of cRNA on ANP32A function with respect to polymerase activity. (A) Schematic representation of wild-type and mutant influenza cRNA promoter structures (5′ promoter C3U + G8A mutations and 3′ promoter A3G + U8C mutations) according to the panhandle model. Red letters indicate mutated residues. (B) Effects of promoter mutations of cRNA on the polymerase activity. (C) Effects of cRNA promoter mutations on ANP32A function with respect to polymerase activity. Error bars represent one standard deviation. (N = 3; ****p < 0.0001, ***p < 0.001, **p < 0.01, and *p < 0.05).

Considering the species-specific regulation of ANP32A in “cRNA to vRNA” replication, we test the effects of promoter mutation of cNA-Luc on the function of ANP32A to regulate polymerase activity. The mutations (3′ promoter A3G + U8C mutations or 5′ promoter C3U + G8A) of cRNA template could stabilize the panhandle structure (Figure 7(A)). The polymerase activity of PR8 virus was analyzed using wild-type or mutant cNA-Luc template. Each type of RNA template was coexpressed with PR8-derived polymerase (PB1, PB2 K627E or 627K, PA) and NP in 293T cells. Polymerase activity were measured 24 h posttransfection. As shown in Figure 7(B), compared with wild-type cNA-Luc, the mutations (A3G + U8C) in the 3′ promoter significantly decreased PB2 K627E polymerase activity but not PB2 627K polymerase activity. The mutations (C3U + G8A) in the 5′ promoter significantly decreased PB2 K627E and 627K polymerase activity, but the difference in both PB2 K627E and 627K polymerases activity were also increased. The high level of chANP32A-X1 did not promote PR8 PB2 K627E polymerase activity with wild-type cNA-Luc template. However, when the mutations (3′ promoter A3G + U8C mutation or 5′ promoter C3U + G8A mutation) of cRNA template were used as a template, coexpression of polymerase (PB1, PB2 K627E or 627K, PA), NP and chANP32A-X1 or huANP32A showed that chANP32A-X1 increased PB2 K627E polymerase activity (Figure 7(C)). The different regulation of chANP32A-X1 in PB2 K627E polymerase activity depended on the cRNA promoter mutation. These observations indicated that the regulation of ANP32A in polymerase activity is likely to occur at the level of the interaction of RNA promoter with the viral polymerase.

### ANP32A has no effect on the primary cRNPs assembly

**Figure 8.**
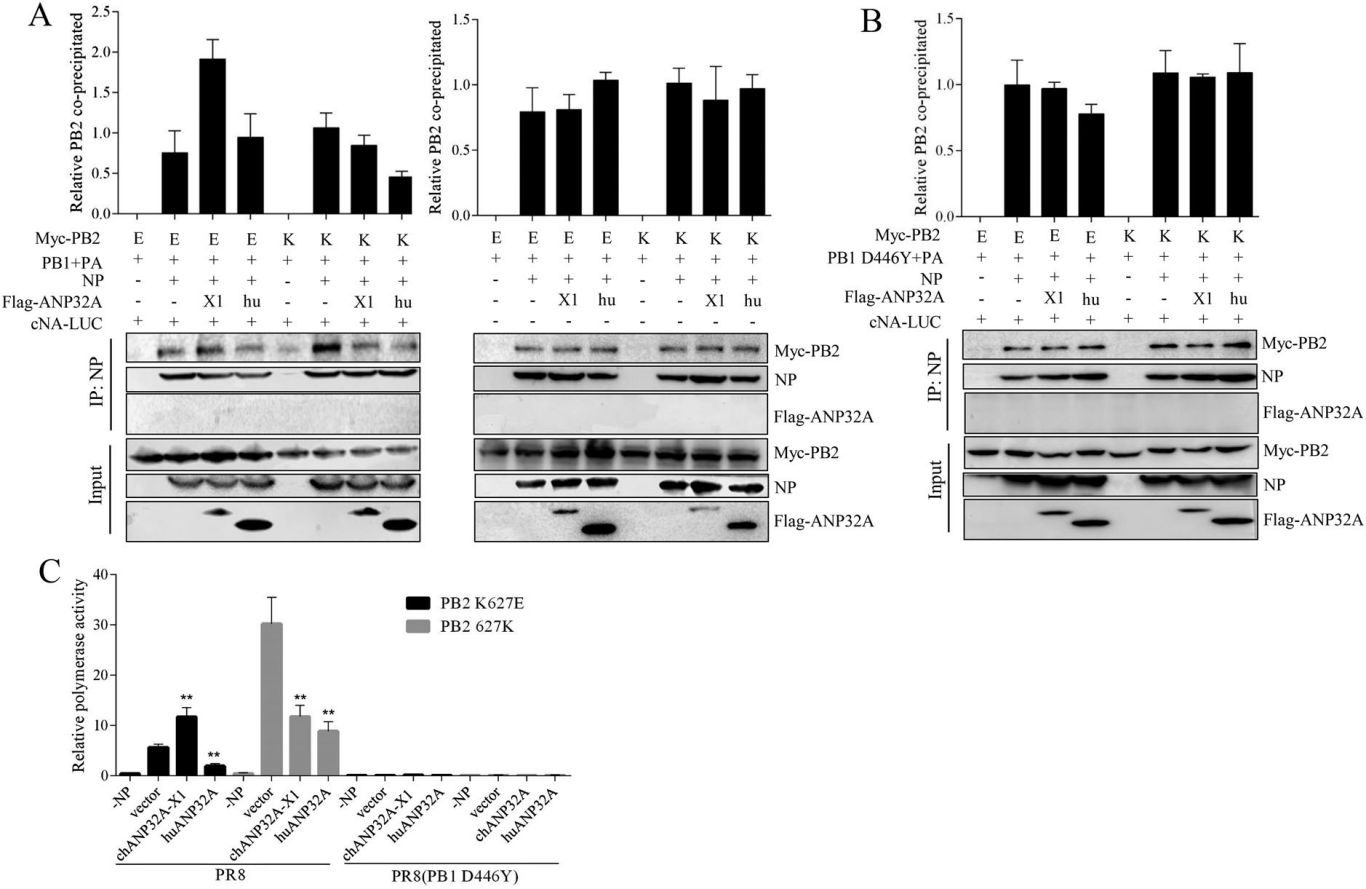
Effects of ANP32A on the amount of NP-precipitated PB2 polymerase in the cRNPs. (A) 293T cells were transfected with PR8 Myc-PB2 K627E or 627K, PB1, PA, and NP as well as ANP32A with or without cNA-Luc. (B) 293T cells were transfected with PR8 Myc-PB2 K627E or 627K, PB1(D446Y), PA, and NP as well as ANP32A with cNA-Luc. The cell lysates were prepared and subjected to NP immunoprecipitation prior to western blot analysis. (C) The polymerase activity from Co-IP samples (A) and (B) was detected. The results shown are the means and standard deviations from three independent experiments. (N = 3; ***p < 0.001, **p < 0.01, *p < 0.05).

The second step of replication of influenza virus, vRNA synthesis from cRNPs, could be differentially regulated by chANP32A-X1 and huANP32A for PB2 627E polymerase in 293T cells; thus, we tested the effects of chANP32A-X1 and huANP32A on cRNPs by coimmunoprecipitation (Co-IP). 293T cells were transfected with PR8 Myc-PB2 K627E or 627K, PB1, PA, and NP as well as cNA-Luc and chANP32A-X1 or huANP32A for 24 h. The cells were harvested for Co-IP and polymerase activity test. NP proteins were detected using a mouse anti-NP monoclonal antibody, while PB2 and ANP32A were detected using an anti-Myc or anti-Flag antibody. We observed that PB2 K627E polymerase exhibited the characteristic defect in RNPs formation and chANP32A-X1 but not huANP32A increased the amounts of PB2 627E in Co-IP, even if in absence of cNA-Luc ANP32A did not affect the amount of PB2 precipitated by NP (Figure 8(A)). When the PB1 D446Y mutation [19] was introduced into the reconstituted polymerase with cNA-Luc, further CoIP assays showed that only cRNPs complex were formed and the amounts of PB2 K627E and PB2 627K polymerase immunoprecipitated by NP were not significantly different (Figure 8(B)). Correspondingly, in polymerase activity detection, chANP32A-X1 that resulted in the increased amount of PB2 K627E precipitated by NP increased polymerase activity and chANP32A and huANP32A could not rescue the inactive catalytic activity of the PB2 K627E and PB2 627K polymerases in PR8 virus with the PB1 D446Y mutation (Figure 8(C)). Collectively, these data demonstrated that chANP32A-X1 is specific for the enhancement of PB2 K627E RNPs formation and that chANP32A and huANP32A did not affect the primary cRNPs (PB2 K627E or 627K) assembly.

## Discussion

ChANP32A, which contains an extra 33aa insertion compared with huANP32A, can restore the mammalian-restricted AIV PB2 627E polymerase activity [24]. Furthermore, the SIM sequence ^176^VLSLV^180^ within the 33 aa insertion is important for promoting PB2 627E polymerase activity [26]. The absence of ^176^VLSL^179^ in the SIM sequence severely impaired the function of chANP32A-X2 to support PB2 627E polymerase activity. In present study, the residues ^180^VK^181^ within the 33 aa insertion are identified to be necessary for ANP32A-mediated different modulation of PB2 627E polymerase activity. These suggested that the entire SIM is required for the optimized function of chANP32A-X1 to stimulate PB2 627E polymerase.

The PB2 627K polymerase had high activity in DF-1 and mammalian cells, but PB2 627E polymerase only restored high activity in 293T cells expressing chANP32A-X1. These data suggested that PB2 627K polymerase effectively utilized chANP32A and huANP32A for its activity, while PB2 627E polymerase was only specific to chANP32A. Knocking out of huANP32A has no effect on human polymerase (PB2 627K) activity or virus growth [27,35,36], but the polymerase activity was abolished in ANP32A and ANP32B double-knockout cells [36], suggesting that PB2 627K polymerase may use huANP32B to support its activity. By contrast, the chicken ANP32B was inactive and did not support avian and human virus polymerase activity [36], thus, only knock out of chANP32A in chicken cells completely ablates virus replication and polymerase activity [37].

Long et al. ruled out the possibility that chANP32A affected the nuclear accumulation of PB2 627E for high polymerase activity in human cells [24]. The huANP32A was reported to be a regulator of vRNA synthesis rather than mRNA transcription of human influenza virus [38], but it was not distinguishable between species-specific regulation of ANP32A on “vRNA to cRNA” synthesis and/or “cRNA to vRNA” synthesis. In present study, we demonstrated that expression of different ANP32A had no species-specific effects on mRNA transcription and “vRNA to cRNA” synthesis, but was species-specific for regulation of “cRNA to vRNA” replication of avian virus (PB2 627E) in 293T cells. The vRNA and cRNA replication complexes are structurally and functionally distinct [15,39] and the replication process of vRNA and cRNA were also different [14,40]. The “cRNA to vRNA” replication has a higher efficiency than “vRNA to cRNA” replication [30]. The differences may lead to regulation of ANP32A in “cRNA to vRNA” replication but not “vRNA to cRNA” replication.

The PB2 E627K mutation or expression of ANP32A_29_ (chANP32A-X2) increased the RNPs (PB2 627E) formation using vRNA template in mammalian cells [25]. Interestingly, we observed that chANP32A-X1 promoted the RNPs (PB2 627E) generation with the cRNA template but ANP32A had no obvious effects on primary cRNPs assembly. The cRNAs produced in virus-infected cells need to be stabilized by newly generated polymerase (PB1, PB2, PA) and NP; otherwise, they become degraded [33]. Thus, the cRNA levels (Figure 5(C)) are also a reflection of the unchanged primary cRNPs assembly. The avian virus (PB2 627E) produced defective cRNPs in human cells, mainly restricting “cRNA to vRNA” replication [34]. In our study, chANP32A-X1 was observed to compensate for the defect in cRNPs, but not in quantitative terms, to promote the production of vRNAs. These data support the restriction of PB2 627E polymerase appears to occur at the earliest steps in the catalytic process after template binding and before elongation, and ANP32A affects these early steps in RNA synthesis [20,25,34]. It is easy to speculate that ANP32A regulates viral polymerase interaction with cRNA promoter for vRNA replication. In addition, the huANP32A did not interact with the polymerase existing in RNPs of human influenza virus [38]. Furthermore, we did not detect the chANP32A-X1 and huANP32A in the RNPs or cRNPs of PB2 K627E or 627K polymerase (Figure 8), suggesting that ANP32A transiently interacted with the trimeric polymerase before the generation of RNPs.

The vRNA 3′ promoter mutations (G3A + C8U) increased vRNA replication of PB2 627E polymerase activity in mammalian cells [20]. Given that ANP32A is species-specific regulation in “cRNA to vRNA” replication, we tested the effects of cRNA promoter mutation on polymerase activity and species-specific regulation of ANP32A. The C3U and G8A mutation at the 5′promoter and the A3G and U8C mutation at the 3′ promoter of cRNA were performed for stabilizing the panhandle structure. The activity of PB2 627E polymerase with the mutant cRNA template was decreased, but could be promoted by the chANP32A-X1, which could not promote in the PB2 627E activity with wild-type cRNA. These observations suggested that promoter mutations of cRNA affected the regulation of ANP32A in PB2 627E polymerase activity. The mammalian-restricted PB2 627E polymerase activity was diminished on short viral templates, which are possible to have a different structure than the long template for effective binding to the PB2 627E polymerase active site [20]. The 3′ promoter mutations (A3G + U8C) or 5′ promoter mutations (C3U + G8A) of cRNA perhaps changing the RNA template structure may lead to uneffective binding to the polymerase active site for decreasing polymerase activity. But ANP32A changes polymerase conformation for effective binding to RNA promoters. Indeed, the influenza virus polymerase required distinct conformational rearrangement of the C terminus of PB2 for promoter binding on vRNA and cRNA [15,16]. The ANP32A regulated “cRNA to vRNA” replication according to our data (Figure 5(C)); thus, they may have a preference for the conformational regulation of the polymerase to make the active site bind to the cRNA promoter. Further structural studies are required to determine the functional conformations of the PB2 after binding to ANP32A.

We raised the model that chANP32A-X1 transiently interacts with the trimeric polymerase and induces a conformational rearrangement of the PB2 627E polymerase that favours the polymerase active site to efficiently and specifically recognize/bind the cRNA promoters for replication initiation.
